# Vitrified Clay for the Production of a Green Sustainable Ultra-High-Performance Fiber-Reinforced Concrete

**DOI:** 10.3390/ma17225624

**Published:** 2024-11-18

**Authors:** Ana Luisa Muñoz-Espinoza, Lucio Guillermo López-Yépez, José Abelardo Valdez-Aguilar, César Antonio Juarez-Alvarado, Alejandro Durán-Herrera

**Affiliations:** Universidad Autónoma de Nuevo León, Facultad de Ingeniería Civil, San Nicolas de los Garza 66455, Nuevo León, Mexico; ana.munozenz@uanl.edu.mx (A.L.M.-E.); llopezy@uanl.edu.mx (L.G.L.-Y.); abelardo.valdezgr@uanl.edu.mx (J.A.V.-A.); cesar.juarezal@uanl.edu.mx (C.A.J.-A.)

**Keywords:** UHPC, vitrified clay, residual flexural strength, flexural toughness

## Abstract

As awareness of the impact of anthropogenic activities on climate change increases, the concepts of durability, resilience, and sustainability in concrete tend to be adopted more seriously in the concrete construction industry. In this sense, one of the concrete technologies that began in the 1980s and that significantly contributes to maximize the beneficial effect on all these concepts are the ultra-high-performance concretes, a very attractive technology because it presents ultra-high strength and durability performances far superior to those of conventional concretes, a performance that is leading to a permanent increased demand. However, the development of these concretes has been widely criticized due to their high ecological impact, which is mainly attributable to the high cement dosages required for their production (800–1000 kg/m^3^). To address this criticism in a comprehensive manner and thereby reduce the embodied carbon attributable exclusively to the material, this research was oriented to determine the effect of an industrial by-product of vitrified clay, as a partial or total substitution for cement, silica fume, and limestone aggregate, on the compressive strength, flexural toughness, and embodied CO_2_. For the UHPC’s evaluated in this work with a dosage of 2% by volume of steel micro-fibers, the results evidence the feasibility that the following substitutions by mass: 30% of the Portland cement, 100% of the silica fume, and 30% of the limestone aggregate and powder, do not detract the fresh stage, the compressive strength, the static modulus of elasticity, and the flexural strength, leading to significant reductions of the embodied CO_2_.

## 1. Introduction

Thanks to the appearance of superplasticizers (1980s) that allowed to produce cementitious matrices with high fluidity and very low water/cement ratios (less than 0.28), the concrete construction industry produced a first generation of concretes, which due to their superior performance in terms of fluidity, consistency, mechanical strength, and durability were called high-performance concretes (HPCs, 1980s), a development that thanks to the potential of these admixtures was preceded by the development of a technology with an even higher performance compared to HPC, which was called ultra-high-performance concrete (UHPC, 1990s), a technology that requires a minimum compressive strength of 120 MPa, with performances in terms of durability, ductility, and toughness far superior to those of conventional concrete [1], a particularity of this mixtures that is attributed to its dense micro-structure and consequent low porosity [2]. Typically, these concretes are produced with a low water/cement ratio (less than 0.25) and are composed of cement (800 to 1100 kg/m^3^), fine silica aggregates (slag, fly ash, ground glass, sand), silica fume (8 to 10% by mass in relation to the weight of cement), admixtures, and steel micro-fibers [3,4]. Coarse aggregates are not used in these concretes because they can cause cracks or micro-cracks that would affect the interstitial transition zone between the coarse aggregate and the cementitious matrix, thus affecting the durability of UHPC [4]. Due to the low water/cement ratios used in their formulations, the cementitious materials do not fully react during hydration reactions, so a significant portion of the cementitious materials will remain as fillers in the cementitious matrix, which leads to high ecological and economic costs [3,4,5,6,7,8,9,10,11,12]. This aspect has been identified by the industry and the international scientific community as an area of opportunity to introduce industrial waste or supplementary cementitious materials to replace mainly the cement, in order to reduce both the economic costs and the ecological costs for these concretes [2,5,6,10,11,12,13]. In this sense, it is also important to highlight that by reducing the dosage of cement in these concretes, we are contributing to a further reduction in the embodied CO_2_ of the material, since the production of Portland cement generates greenhouse gases emissions that range between 5–8% of the total emissions emitted by anthropogenic activities [5,14,15].

On the other hand, also with the objective of complementing the efforts to reduce the carbon footprint attributed to concrete, significant efforts are being made internationally to reduce the exploitation of natural resources required for the production of cement and concrete by searching for industrial residues or by-products, such as rice husk ash, fly ash, ground glass dust, and granulated blast furnace slag, to be used as supplementary cementitious materials in concrete [11,16,17,18,19]. Due to its availability in the earth’s crust, a significant number of research works have been published in recent decades, aimed at evaluating the incorporation of calcined clays in cement production [20,21], as well as waste from the ceramic industry that have been used as aggregates in conventional and high-strength concretes [12,22,23]. Because of their important contributions to the packing and densification of the cementitious matrix, as well as to durability, several papers have been published over the last three decades on the effect of various supplementary cementitious agents on rheological, mechanical, yield strength, and durability performance when incorporated in a UHPC [11,13,17,24,25,26].

In Mexico, an industrial waste that, due to its chemical and mineralogical composition, represents a viable option for its utilization in the formulation of Portland cement-based hydraulic concretes is vitrified clays. In 2017, the generation of these wastes and others such as glass, sands, demolition materials, among others, reached 120,128 tons [27], which was considered as municipal solid waste and was sent to landfills. This study has the objective of contributing to the optimization of new UHPC formulations, by substituting several of their ingredients with vitrified clay in order to reduce their economic and ecological cost, as well as their negative impact on sustainability, which will also positively reduce the presence of particles smaller than 10 microns in the air that is breathed in by the populations surrounding the aggregate banks, since in many cases they are above the limits allowed by the urban regulations, causing consequently health problems to the populations in the large cities.

In the extensive bibliographical review that was carried out to demonstrate the relevance and need for this work, it was identified that in the production of UHPC, the most used materials in substitution of the cement, are fly ash, granulated blast furnace slag, rice husk ash, and glass powders, but no papers were found reporting work on UHPC with vitrified clay. The results reported in this work show the potential of vitrified clay as a viable option to replace cement and silica fume in the production of a UHPC concrete, without significantly detracting from properties such as fresh stage consistency, consistency permanence, compressive strength, static modulus of elasticity, modulus of rupture, and residual flexural strength after the first crack. On the other hand, the substitution of cement, silica fume, and limestone sand/powder by vitrified clay leads to a significant reduction in the embodied CO_2_.

## 2. Materials and Methods

### 2.1. Materials

The following particulate materials were used for the production of the UHPCs involved in this work: an ASTM C150 [28] Type II Portland cement (CPO); a condensed silica fume (SF) with an ASTM C1240 [29] strength activity index of 97%; a limestone powder (LP, D_50_ = 46.3 μm) obtained as a by-product of the concrete aggregates production; an ASTM C33 [30] limestone sand (LS, D_50_ = 950 μm); a vitrified clay (VC) that was grinded to obtain the particle size required by ASTM C33 for a fine aggregate (VC, D_50_ = 950 μm), and was further pulverized-micronized to obtain three additional fractions of vitrified clay with average particle sizes of D_50_ = 46.3 μm to substitute for the limestone powder, D_50_ = 15.2 μm with an ASTM C311 [31] strength activity index of 92% to substitute for the cement, and D_50_ = 8.4 µm with an ASTM C1240 pozzolanic activity index of 105% to substitute for the silica fume.

A polycarboxylate-based superplasticizing admixture was used in the dosage necessary to obtain the target consistency of 325 ± 25 mm, without compaction with the tamper or compaction by the free fall of the flow table, as indicated in the standard procedure of ASTM C 1437 [32]. Because the initial mixtures presented high contents of entrapped air (7–8%), in order to maximize the level of densification of the cementitious matrix it was necessary to incorporate a defoamer or air-occluding admixture, Isodense 250, produced by CEMEX Admixtures. In the fiber-reinforced concretes, a Bekaert DRAMIX OL 13/.20 micro-steel fiber made of cold-drawn wire, composed of filaments with a diameter of 200 µm and a length of 13 mm, was used. Table 1 shows the properties of the particulate materials and Table 2 the included particle size distribution of the particulate materials used to produce the UHPCs.

### 2.2. Optimization of Mixture Proportions

**Figure 1:**
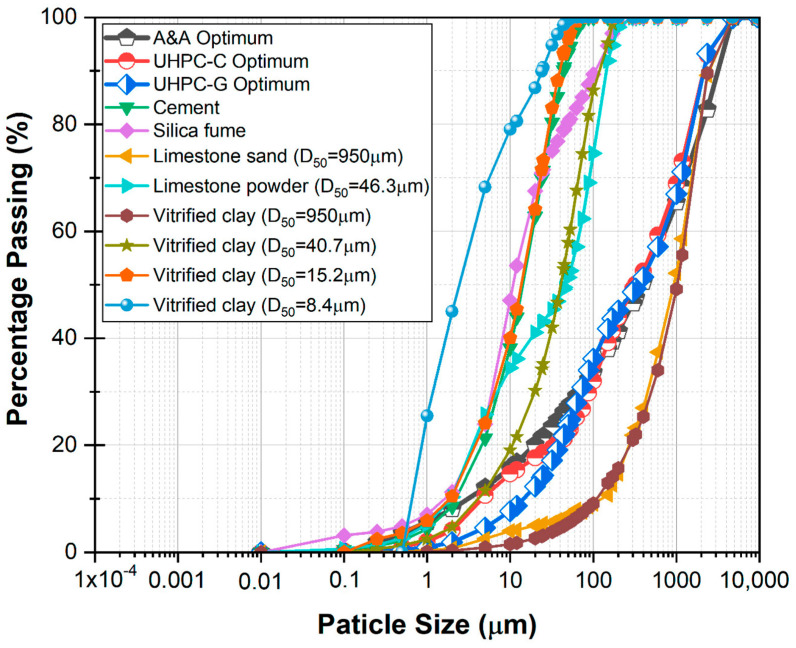
Particle size distribution of cement and silica fume. Andreasen and Andersen’s best particle packing optimization (A&A Optimum) and best particle packing optimizations for UHPC-C and UHPC-G.

**Figure 2:**
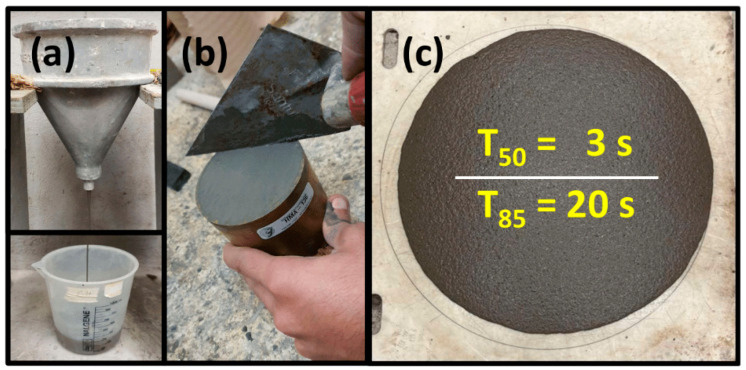
Fresh stage characterization: (**a**) saturation points through the ASTM C939 flow cone, (**b**) air content, and (**c**) slump flow.

To maximize the granular packing or in other words the level of densification of the composite in both the green and reference concrete and thus minimize the void volume, the equation proposed by Andreasen and Andersen, shown in Figure 1 as A&A Optimum, was used [11,33]. The particle packing optimization was further improved through the incorporation of the air-occluding admixture, leading to a decrease in air content from an average of 7.5% to 3.2% for the reference concretes to 2.3% for the green concretes without fibers and to 1.5% for the green concretes with fibers. The optimal dosage of superplasticizer, known as the saturation point, was determined for the paste fraction of UHPC-C through the flow cone and the standard procedure described in ASTM C939 [34] (Figure 2a). The reference concrete (UHPC-C) was prepared with Portland cement and silica fume as cementitious materials, limestone sand as aggregate, and limestone powder obtained as a by-product of the manufacture of concrete aggregates as filler. For the production of the green concrete (UHPC-G), the vitrified clay was crushed and pulverized to obtain four different fractions with particle size distributions similar to those of the particulate materials with which the reference concrete was produced, and which were dosed in the green concrete to maximize the substitutions of limestone sand (fraction of vitrified clay with D_50_ = 950 µm), limestone powder (fraction with D_50_ = 46.3 µm), cement (fraction with D_50_ = 15.2 µm), and silica fume (fraction with D_50_ = 8.4 µm).

For both concretes, the water/cement ratio was 0.25, the cementitious dosage was 800 kg/m^3^, and the target consistency was 325 ± 25 mm, measured by the flow table specified in ASTM C230 [35]. Green concrete with optimized proportions in terms of compressive strength and consistency led to a substitution of 30% of limestone sand by vitrified clay with D_50_ = 950 µm, 30% of limestone powder by vitrified clay powder with D_50_ = 40.7 µm, 30% of Portland cement by vitrified clay powder with D_50_ = 15.2 µm, and 100% of silica fume by vitrified clay with D_50_ = 8.4 µm. The mixture proportions for the four UHPCs evaluated in this work are presented in Table 3.

### 2.3. Test Methods 

#### 2.3.1. Characterization of Cementitious Materials, Fine Aggregates, and Powders

The particle size distribution for the particulate materials to be used as fine aggregates in the production of the concretes involved in this research work (limestone sand and vitrified clay sand with D_50_ = 950 µm) was determined according to the standard procedure described in ASTM C136 [36] and the specific gravity and water absorption through the standard procedure described in ASTM C128 [37]. Specific gravities for cement, silica fume, limestone powders, and vitrified clay powders were determined following the procedure described in the standard test method ASTM C604 [38]. Water absorption values for sands, limestone powder (D_50_ = 46.3 µm), and the vitrified clay powder (D_50_ = 40.7 µm) were determined following the procedure described in ASTM C128. Particle size distributions were determined using a Malvern Panalytical Mastersizer 2000, Malvern, England, particle size analyzer, and the strength activity indexes for the silica fume and the fine vitrified clay powder (D_50_ = 8.4 µm) were determined according to the procedures described in ASTM C1240 and ASTM C311, respectively.

#### 2.3.2. Fresh State Properties of UHPCs

For the four UHPCs, the fresh concrete properties characterization included: unit weight and air entrained content according to the procedure described in ASTM C185 [39]; setting times by penetration resistance according to the procedure described in ASTM C403 [40]; extensibility and loss of consistency determined as the average of two perpendicular readings, with the cone described in ASTM C 1437 [32] on a rigid laboratory table with smooth surface. The loss of consistency was measured every 15 min after the first extensibility measurement; for this purpose, prior to each measurement, concrete was left to rest inside the bowl and then re-mixed at medium speed for 30 s. The corresponding slump flow, and the relative viscosity measured as the time it takes for the outer edge of the concrete mass to reach a diameter of 500 mm and 850 mm, were determined according to the standard procedure described in ASTM C1611 [41]. 

Figure 2a shows the ASTM C939 flow cone used to determine the optimum dosage for the superplasticizer admixture (saturation point), and Figure 2b shows the final stage of the standard procedure followed to determine the air content in the different concretes. Figure 2c shows the appearance of UHPC-C and the times taken for this concrete to reach extensibility of 500 and 850 mm.

#### 2.3.3. Mechanical and Elastic Properties in the Hardened State

To determine the compressive strength, 50 mm cubes were made in bronze molds according to the procedure described in ASTM C109 [42], and tested in a Toni Technik hydraulic press, model ToniNORM 2031/ToniPRAX, series 1543, Berlin, Germany, with a maximum capacity of 2000 kN, at the ages of 1, 3, 7, 28, 56, and 91 days. For the determination of the static modulus of elasticity, three specimens with a diameter of 10 cm and a height of 20 cm were prepared according to the procedure described in ASTM C192 [43]. After preparation, the specimens were stored under standard curing conditions (T = 23.0 ± 2.0 °C, RH = ≥ 95%), and then subjected to compression tests in the Toni Technik press, model ToniNORM 2031/ToniPRAX, series 1543, according to the standard procedures described in ASTM C39 [44] and ASTM C469 [45].

**Figure 3:**
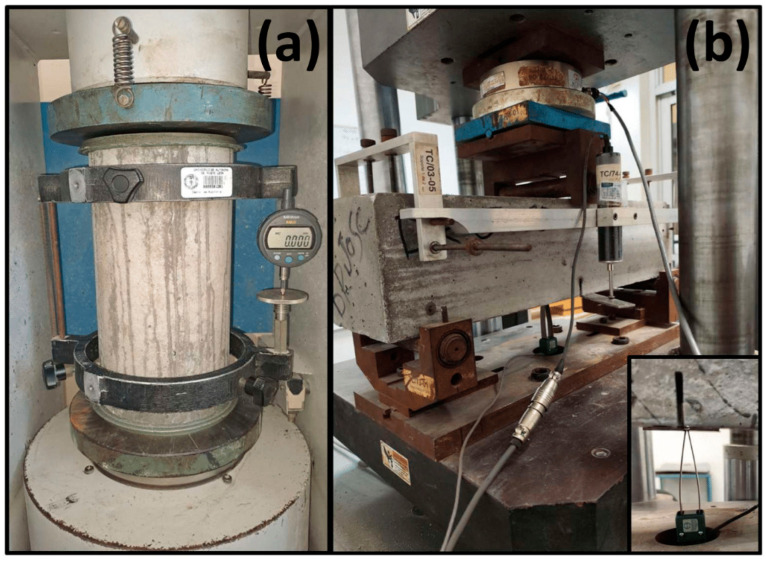
Setups for the determination of the static modulus of elasticity (**a**), and for the determination of the modulus of rupture and post-cracking residual strengths (**b**).

For the four concretes, the preparation of the specimens and the mechanical-elastic bending tests on beams with dimensions of 15 × 15 × 60 cm, cured under standard conditions and tested at the age of 28 days, were carried out according to the procedure described in ASTM C78 [46], to determine the modulus of rupture of concretes without fibers (UHPC-C and UHPC-G), and according to the procedure described in the standard UNE-EN 14651-2007 [47] to determine the residual strength after cracking. Before the bending test, in each beam a cut of 20 mm deep and 3 mm wide was made with a diamond disk across the width of the beam and in the center of the span. At the age of 91 days, the specimens were subjected to bending tests in an INSTRON universal machine, Model 600DX, Norwood, MA, USA, with a capacity of 60 tons. During the bending tests, the measurement of the progressive crack opening widening (CMOD) was performed by means of an Epsilon model 3541 clip-on deformimeter, Atlanta, GA, USA, and a National Instruments data acquisition unit (DAQ), which during the test was linked to a computer by means of the LabVIEW 2013 software Austin, TX, USA. The residual strengths were calculated using the load-deflection/deformation curves and the equations provided in Sections 9.2 and 9.3 of the UNE-EN 14651-2007. The test setups to determine the static modulus of elasticity, the modulus of rupture, and the post-cracking residual strengths are presented in Figure 3.

The stresses at the first crack (LOP, limit of proportionality) and the residual strengths, were calculated with Equations (1) and (2) that were adopted from Sections 9.2 and 9.3 of UNE-EN 14651-2007, respectively:

(1) $$f_{LOP}=\frac{3F_{L}l}{2bh_{sp}^{2}}$$

(2) $$f_{r}=\frac{3F_{J}l}{2bh_{sp}^{2}}$$

Where:

$f_{LOP}$ = Stress in the limit of proportionality, N/mm^2^.
$f_{R,j}$ = Residual flexural tensile strength, corresponding with *CMOD* = *CMOD_j_* or $\delta =\delta_{j}\left(j=1,2,3,4\right)$ N/mm^2^, where the corresponding CMODs are 1 = 0.5 mm, 2 = 1.5 mm, 3 = 2.5 mm and 4 = 3.5 mm.
  $F_{L}$ = Corresponding load for the LOP, N.
$F_{j}$ = Corresponding load for CMOD = CMODj or δ = δj (j = 1, 2, 3, 4), N.
l = Length of span, mm.
b = Specimen length, mm.
$h_{sp}$ = Distance between the notch tip and the top of the sample, mm.

## 3. Results and Discussion

### 3.1. Fresh State Properties

The volumetric weights of the reference concretes (UHPC-C and SFR-UHPC-C) in fresh stage were 2400 kg/m^3^ on average, and those of the green concretes (UHPC-G and SFR-UHPC-G) were 2300 kg/m^3^ on average. The difference of 100 kg/m^3^ is due to the lower density of the different fractions of the vitrified clay in relation to the ingredients substituted in the green concrete optimization process (limestone sand, limestone powder, cement, and silica fume). The air contents suggest an improvement in the densification of the green concrete due to the packing provided by the particle size distributions of the different vitrified clay fractions used in the optimization of the green concrete, since for the reference concretes (UHPC-C and SFR-UHPC-C) the air contents were between 3.24 and 3.22%, and for the green concretes (UHPC-G and SFR-UHPC-G) the air contents were between 2.29 and 1.50%, respectively. In terms of densification, the presence of fibers had no effect on the reference concrete, but it did have an effect on the green concrete, as it resulted in a 0.79% decrease in air content, which is attributed to the higher viscosity/cohesiveness perceived by the naked eye for the green concrete, compared to the reference concrete, which apparently resulted in a lower amount of voids at the fiber–cement matrix interface and, consequently, a lower amount of air entrained.

Flowability is a characteristic of great importance, since it also reflects the stability of the mix to ensure the absence of segregation, allowing us to guarantee correct placement, proper setting, and the best possible finish, an aspect that is highly valued in architectural applications.

**Figure 4:**
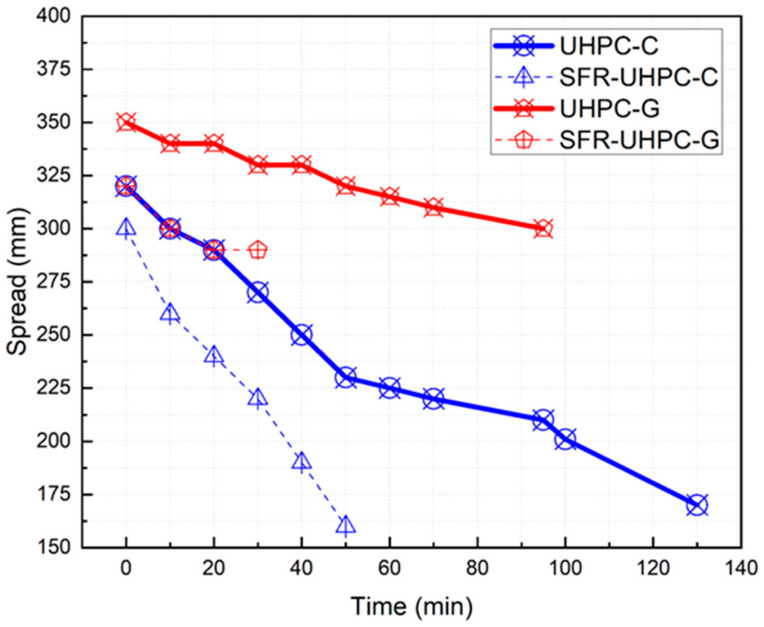
Loss of extensibility over time.

To achieve the target flowability of 325 ± 25 mm, the dosage of the superplasticizer admixture resulted in a flowability of 320 mm for the reference mixture (UHPC-C) and 350 mm for the green concrete (UHPC-G). The dosage of the fibers resulted in a flowability reduction in both concretes. This reduction is attributed to the high fiber concentration (2% by volume) and to the high specific surface area of the filaments that make up the fiber, since they are very fine compared to the geometry of conventional fibers. Figure 4 shows the loss of consistency of the four concretes evaluated in this work, where we see that the reference concrete shows a more pronounced loss of consistency than the green concrete, as well as the fact that this concrete allowed the measurement of the consistency even up to 2 h:10 min after mixing, and the green concrete only up to 1 h:35 min after mixing, which we attribute to two factors: the higher reactivity of the cementitious compounds (Portland cement and silica fume), and the high fineness and high reactivity of the silica fume. In the case of the concrete with fibers, it was observed that the loss of consistency was more pronounced in both concretes, and that the measurement of this property was possible for the reference concrete (SFR-UHPC-C) up to 50 min after mixing, and for the green concrete (SFR-UHPC-G) up to 30 min after mixing. The results shown in Figure 4, which represent the permanence of consistency for the four concretes, clearly indicate the times after which it was no longer possible to measure the consistency; in this sense, the reference concretes allowed measuring the consistency up to an average extensibility of 160 mm and in the case of the green concretes up to an average extensibility of 315 mm. The higher cohesiveness exhibited by the green concrete results in Figure 4 is attributed to the fact that, most likely, all the fractions of vitrified clay that were incorporated in the substitution of limestone powder, limestone sand, cement, and silica fume are composed of more angular particles, which at the same time have a rougher surface.

For practical applications, it is desirable that UHPCs maintain their high flowability for an appropriate period to facilitate the placement and accommodation of the concrete within the formwork, and that the setting times allow these operations to be carried out in the best possible way, without affecting the development of the mechanical, microstructural, and architectural properties. In this sense, the setting times were not very different from those obtained for a conventional concrete, with initial setting times of 3 h:52 min for the reference concrete (UHPC-C) and 3 h:17 min for the green concrete (UHPC-G), as well as final setting times of 5 h:02 min and 5 h:08 min, respectively, suggesting that three hours are available for placing and setting. However, this is not the case since, as we can see in Figure 4, because of the rate of consistency loss, the reference mixtures would only provide a period of about 20 min to perform these activities properly, and in the case of the green concretes, this period could be extended to 40 min, times that are reasonable for the precast industry.

### 3.2. Mechanical and Elastic Properties of UHPC in the Hardened State

#### 3.2.1. Compressive Strength 

**Figure 5:**
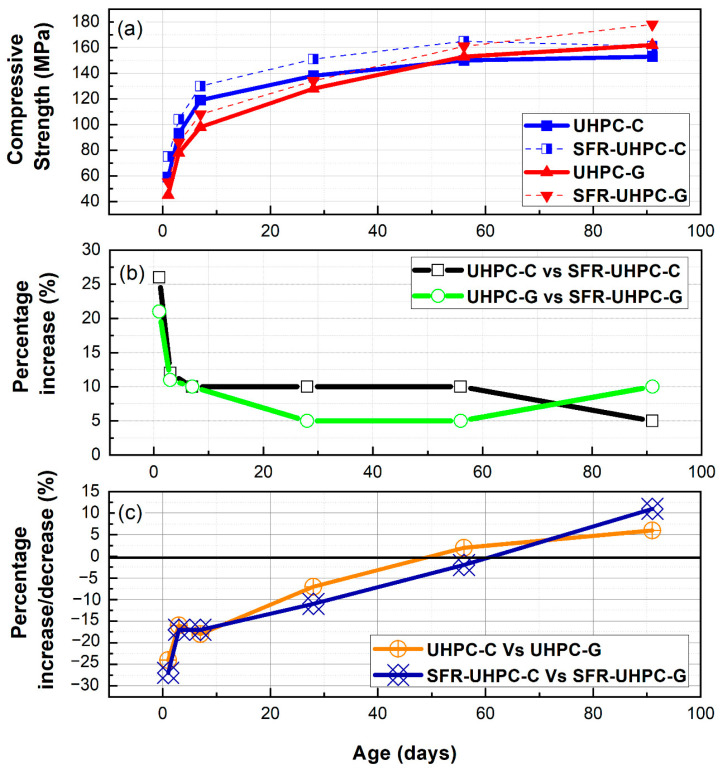
(**a**) Compressive strength development for mixtures UHPC-C, SFR-UHPC-C, UHPC-G, and SFR-UHPC-G; (**b**) compressive strength increase attributed to the presence of steel micro-fibers; and (**c**) increase/decrease of compressive strength resulting from the substitutions of cement, silica fume, limestone powder and sand, by vitrified clay.

According to the standard practice to cast and test UHPC specimens, that is detailed in ASTM C1856 [48], for concrete to be classified as a UHPC, it must have a minimum compressive strength of 120 MPa [1,11]. The compressive strength development for the four concretes evaluated in this work, for ages ranging from 1 to 91 days, is shown in Table 4 and Figure 5a. The slopes that appear in Table 4 for four different time windows are indicative of how active the cement hydration reaction is at early ages and how significant the pozzolanic reaction of the vitrified clay is at later ages. The four concretes exceed 120 MPa with respect to the strengths obtained at the age of 28 days in standard curing, and the strengths of the reference concretes increased by 11% (UHPC-C) and 6.6% (SFR-UHPC-C) at the age of 91 days. For the green concretes, these increases were significantly higher, with values of 27% (UHPC-G) and 33% (SFR-UHPC-G), which can be attributed to the pozzolanic reaction of the vitrified clay, an aspect that is also clearly illustrated by the average increase in strength of the green concretes (UHPC-G and SFR-UHPC-G) between the ages of 7 and 28 days, with strength increases of 1.4 and 1.2 MPa/day, respectively, although the four concretes showed similar strength gains between 1 and 7 days (8.8 to 10.0 MPa/day). For the green concretes, the significant increase in strength due to the pozzolanic activity of the vitrified clay is clearly evident for the following three time periods: between 1 and 7 days, between 7 and 91 days, and between 28 and 91 days, when the strength development of the green concretes was 20 to 55%, 85 to 212%, and 225 to 438% higher, respectively, than that of the reference concretes.

Although there is no precise point of separation between high-strength concrete and normal-strength concrete, the American Concrete Institute defines high-strength concrete as one with a compressive strength greater than 41.4 MPa, a strength that the four concretes evaluated in this work present before an age of 24 h. This significant aspect is of major importance for the precast industry, since the cycle for reusing the molds would be very short, a feature that is very attractive for this sector of the industry.

For the different ages at which the compressive strength was experimentally determined, the values used to construct the two graphs that appear in Figure 5b were obtained by calculating the percentage increase/reduction attributable exclusively to the presence of the fibers, that were obtained using the following equations: [(UHPC-C) − (SFR-UHPC-C/UHPC-C)] × 100, the results of which are represented by the black curve, and [(UHPC-G) − (SFR-UHPC-G/UHPC-G)] × 100, the results of which are represented by the green curve. Similarly, Figure 5c shows the percentage of increase/reduction attributable exclusively to the influence on the hydration and/or pozzolanic reactions of the vitrified clay, that were obtained with the following equations: [(UHPC-C) − (UHPC-G/UHPC-C)] × 100, whose results are represented by the orange curve, and [(SFR-UHPC-C) − (SFR-UHPC-G/SFR-UHPC-C)] × 100, whose results are represented by the blue curve.

As can be seen in Figure 5b, for the reference and green concretes, at the age of 1 day, the incorporation of steel fibers originated significant gains in terms of compressive strength, with increases of 27% (SFR-UHPC-C vs. UHPC-C) and 22% (SFR-UHPC-G vs. UHPC-G), a gain that after 7 days remained between 5 and 10% (SFR-UHPC-G vs. UHPC-G).

Figure 5c illustrates the effect of vitrified clay as a replacement for limestone sand, limestone powder, cement, and silica fume, in the percentages resulting from the optimization process. In this figure, it is clear that there was a gain of strength at the age of 1 day (24–27%) and that for the time interval between 3 and 58 days, the gain in strength for green concretes was more aggressive in relation to the reference concretes, exceeding from 50–58 days the strength of the reference concretes, to conclude with increments of 6% (UHPC-G) and 11% (SFR-UHPC-G) at the age of 91 days. Compared to the reference concretes, the upward trend in the compressive strength of the green concretes is attributed to the pozzolanic reactivity and the high hardness of the vitrified clay [13,49].

#### 3.2.2. Static Modulus of Elasticity at 91 Days

The static modulus of elasticity for the reference concretes with and without fibers (UHPC-C and SFR-UHPC-C) were 34.7 and 33.5 GPa, and for the green concretes were 39.7 and 35.2 GPa, respectively. Comparing these results, we observed that the green concretes without fiber and with fiber showed results higher than the reference concretes by 15.0 and 5.0%, respectively, which could be attributed to the better granular packing suggested by the lower air content, to a better mechanical interlock due to the angular morphology of the vitrified clay particles, and to the pozzolanic activity of this material. The results also illustrate that for the reference concrete the presence of fibers originates a reduction of 3.4% in the modulus of elasticity and of 11.3% for the green concrete, which is attributed to the fact that the adhesion capacity of the cementitious matrix is weakened at the interface between the matrix and the surface of the fibers, since the surface of the filaments is smooth. In the case of green concrete, the reduction is more significant, since in green concrete the cementitious matrix has a weaker cementitious potential compared to the cementitious matrix of the reference concrete, due to the higher cementitious potential of cement and silica fume compared to the vitrified clay fractions with which these materials were replaced in green concrete. The results obtained are consistent with the results previously reported for the static modulus of elasticity for ultra-high-performance concretes, between 30.0 and 45.9 GPa [7,50,51].

#### 3.2.3. Modulus of Rupture, Post-Cracking Residual Strengths, and Toughness Indexes

For the concretes without fiber, the average modulus of rupture was 5.2 MPa for the reference or conventional concrete (UHPC-C) and 5.1 MPa for the green concrete (UHPC-G). These results are the average of three specimens that, after being demolded 24 h after fabrication, were kept under standard curing conditions until their testing age at 28 days. As a parameter of the uncertainty in the measurements, the coefficients of variation resulted in values of 21% and 18%, respectively.

The post-cracking residual strengths were determined by flexural testing of the fiber-reinforced beams, which in their preparation were slotted with a diamond saw at the center of the span, according to the procedure described in UNE-EN 14651-2007, for which the load values and the corresponding progressive crack mouth opening displacements (CMODs) were measured, with which the corresponding displacements/deflections were subsequently calculated by means of the equation δ = 0.85CMOD + 0.04, provided in this standard, where δ = displacement/deflections in millimeters.

##### Post-Cracking Behavior

**Figure 6:**
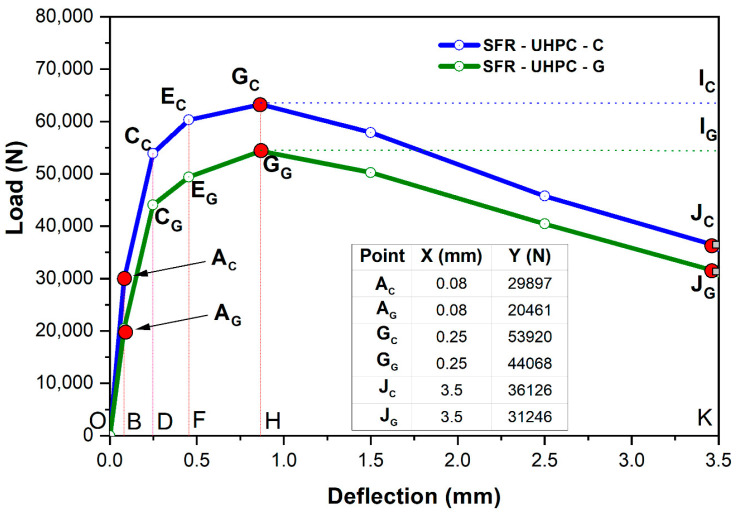
Bending strain development of fiber-reinforced concrete beams, showing the significant post-cracking capacity attributable to the presence of the steel micro-fibers.

In the construction industry, hydraulic concrete is a material that can now be designed for compressive strengths between 20 and 200 MPa, or higher. This particular property is one of the strengths of the material, for the many different applications in which it is used; however, one of its major weaknesses is its tensile strength, which is often counteracted using steel bars, FRP bars, and metallic or synthetic fibers. Due to the high strength of UHPC, its cementitious matrix offers a much better adhesion with all these reinforcements, so the performance of the reinforcement is optimized, as is the case of the fibers in the UHPC, a benefit that can be clearly observed in Figure 6. 

In the stage of residual stresses that occurs after the concrete presents the first crack, two types of behavior can be observed, the one known as strain softening, which is typical in conventional fiber-reinforced concretes, and the one known as strain hardening, which is typical in high-strength concretes [52]. The fracture mechanics involved in these behaviors depend on various factors specific to the fibers, such as the type of fiber, the dosed volume, the aspect ratio, and the adhesion of the fiber to the cementitious matrix. In this sense, since the concretes studied in this research work are ultra-high-strength concretes, in the load-deflection behavior presented graphically in Figure 6, as a result of the flexural test of the beams, the fiber-reinforced concretes SFR-UHPC-C and SFR-UHPC-G exhibit a clear strain hardening (segments AC-GC and AG-GG, respectively), followed by a clear strain softening (segments GC-JC and GG-JG, respectively).

During strain hardening (0.08 mm ≤ δ ≥ 1.0 mm, segments AC-GC and AG-GG, respectively), the acting tensile stresses exceed the cracking resistance of the concrete cementitious matrix, leading to the appearance of multiple micro-cracks, until reaching the deflection threshold of 0.87 mm, from which the adhesion between the fiber filaments present in the plane of the crack and the cementitious matrix starts to be lost, leading to a progressive decrease in the load capacity and to the consequent appearance of a localized main crack, which in our case was induced by the slot made in in the center of the span of each beam [52].

The three stages clearly distinguished in Figure 6 illustrate the benefit in terms of ductility to which the incorporation of the steel fibers used in this research leads: Stage I—appearance of the first crack (points AC and AG), Stage II—strain hardening (stage between points A and G), and Stage III—strain softening (stage between points G and J). For all these stages, the conventional reference concrete with fibers (SFR-UHPC-C) exhibits the best performance, which is exclusively attributed to a better bonding capacity between the cementitious matrix and the surface area of the fibers. For these three stages, the green fiber-reinforced concrete (SFR-UHPC-G) shows reductions of 7.69%, 13.94%, and 17.69%, compared to SFR-UHPC-C. This lower performance is attributed to the fact that the cementitious matrix in the green concrete has a lower bonding capacity in comparison to the cementitious matrix in the reference concrete.

#### Proportional Limits and First Crack Stresses

**Figure 7:**
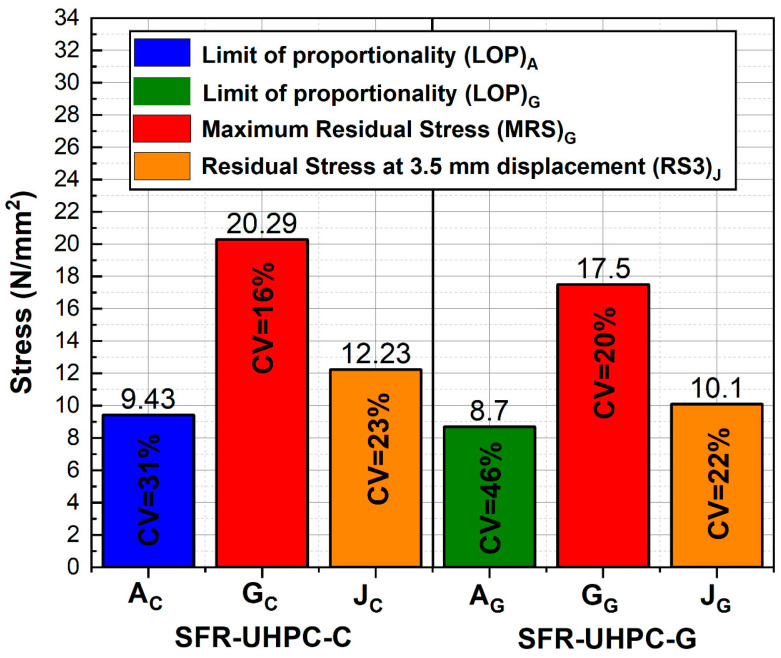
Limits of proportionality, residual strengths, and coefficients of variation for three specimens.

For fiber-reinforced concretes (SFR-UHPC-C and SFR-UHPC-G), the flexural tensile strengths or limits of proportionality (LOPs) were measured by flexural testing of the slotted beams with center load. The LOPs corresponding to the appearance of the first crack and to the end of Stages II and III, which correspond to the residual strengths at the end of strain hardening and strain softening stages, respectively, are shown in Figure 7. In this figure we can notice that the reductions reported in terms of load in Section Post-Cracking Behavior correspond to the following reductions: 2.8, 2.91, and 1.45 MPa in terms of flexural strength. As a parameter of the measurement uncertainty for the strengths corresponding to the appearance of the first crack, the coefficients of variation resulted in values between 16% and 31% for SFR-UHPC-C, and between 20% and 46% for SFR-UHPC-G. As we can see, the CVs present very high values and vary significantly among them, which is mainly attributed to the distribution of the fiber filaments in the cementitious matrix, and to the orientation of the filaments and the amount of fiber in the failure plane where the induced crack is located.

For both concretes, Figure 7 illustrates the great benefit of incorporating the fibers, since in the strain hardening stage (LOPG), the presence of the fibers led to a significant increase in the residual strength in comparison to the ones obtained for the first crack (LOPA); the values of 10.86 MPa (115.16%) and 8.80 MPa (101.15%), for SFR-UHPC-C and SFR-UHPC-G concretes, respectively, increase so that despite being reduced during the strain softening stage (LOPJ), the residual stress remains above the stresses corresponding to the first crack (LOPA) by 2.80 MPa (29.69%) and 1.40 MPa (16.09%). The results presented in this graph also illustrate the reductions caused by the substitution of vitrified clay for the different fractions of cement, silica fume, limestone powder, and limestone aggregate, which were 7.74% for the appearance of the first crack, 13.75% for the end of the strain hardening stage, and 17.42% for the end of the strain softening stage.

#### Flexural Toughness Indexes

The toughness of cement concrete can be divided into bending toughness, impact toughness, and fracture toughness. In the case of bending toughness, the material resistance to breaking apart represents the energy absorbed which is called the flexural toughness, being the area under the load-deflection curve for the concrete prism subjected to a four-point bending test. This energy can show to what extent the fiber-reinforced concrete can withstand loading conditions until broken. Accordingly, the additional energy which must be consumed to lead to either fiber pull-out or fiber rupture enhances the toughness and post-peak behavior (strain softening) of the matrix [53].

For the fiber-reinforced concretes in this work, the flexural toughness was determined by quantifying the area under the stress-strain curve obtained through flexural tests on beams, and the toughness indexes were also determined. These toughness indexes are defined as the relationship between the energy absorption capacity in the residual stage, with respect to the energy absorbed in the pre-cracking stage (appearance of the first crack). In this research, to characterize the fiber-reinforced concretes, the toughness indexes I_5_, I_10_, and I_20_ were calculated, which according to ASTM C1018-97 [54] correspond to the numbers obtained by dividing the area up to a deflection of 3, 5.5, and 10.5 times the first crack deflection by the area up to first crack, and the ultimate toughness index I_r_, corresponding to a deflection of 3.5 mm.

**Figure 8:**
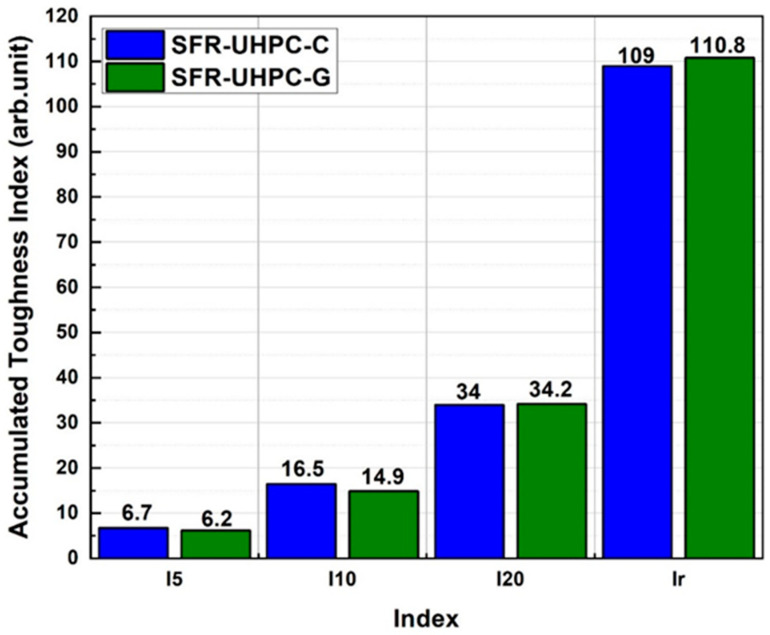
Accumulated toughness Indexes (Arb. Units = dimensionless units).

Figure 8 shows that in all cases the toughness indexes for SFR-UHPC-C and SFR-UHPC-G concretes are very similar or practically the same. Considering for both concretes average values of 6.0, 15.5, and 34.0, for the toughness indexes I_5_, I_10_, and I_20_, it can be observed that these are higher than the maximum values reported in Table 5 (5.7, 12.9, and 29.0, respectively), which in terms of flexural toughness indicates that the synergistic effects of the fibers and the cementitious matrix used in this work are congruent with those reported in previous works, as well as presenting a greater energy absorption capacity under bending loads. For this work, the deformation corresponding to I_r_ (3.5 mm) represents 43.75 times the deformation at which the first crack occurred (0.08 mm); although this deformation is probably too high for the functionality and operation of a building, the average flexural toughness (I_10_) shows a residual capacity for bending energy absorption of 223.53% higher than the average for I_20_, an aspect that undoubtedly contributes to improving the structural safety of a specific building.

**Figure 9:**
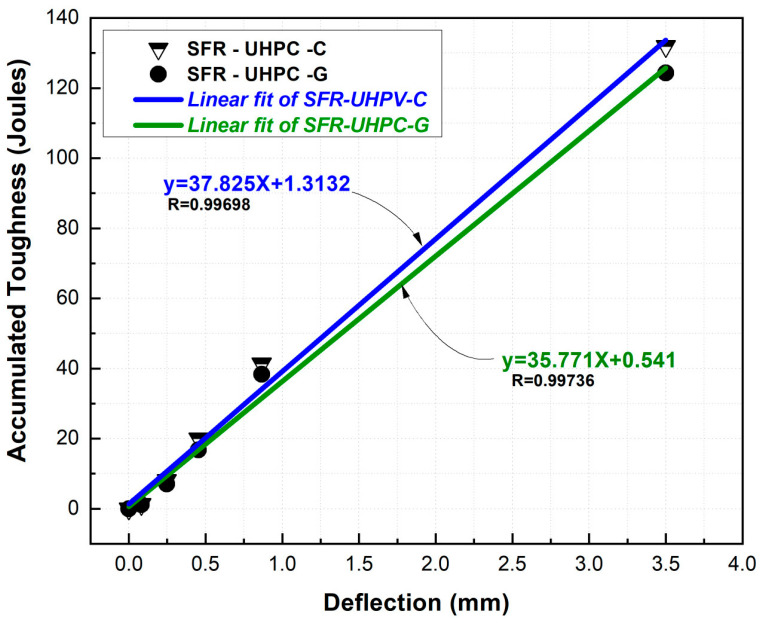
Linear fits of accumulated toughness.

The results reported in Figure 8 were used to establish the linear regressions presented in Figure 9, which can be very useful in practice, since, for a particular project, they offer the user the possibility of estimating the cumulative flexural toughness for a maximum allowable deflection. The linear regressions presented in this graph indicate a higher cumulative flexural toughness development of 2.054 Joules/mm deflection for SFR-UHPC-C concrete relative to SFR-UHPC-G concrete. For a deflection of 1 mm, values for accumulative toughness in the range of 31 to 39 Joules have been reported [57], which are congruent with those obtained for the concretes evaluated in this work, 39.14 Joules for the SFR-UHPC-C concrete and 36.31 Joules for the SFR-UHPC-G concrete.

Although the results and uncertainty indicators reported in this work were obtained for experimental laboratory tests, these exhort us to use vitrified clay at an industrial scale in a sector with very strict controls that could be most benefited in the first instance, the precast concrete industry. When transferring them, the differences in relation to the results and uncertainty indicators are expected to be minimal or negligible.

### 3.3. Estimation of Embodied CO_2_

In the design of future building materials, in addition to prioritizing engineering properties such as compressive strength, flexural strength, ductility, and toughness, it is imperative that they are optimized in terms of their carbon footprint, so that the material contributes to increasing the favorable impact of the material on the sustainability of anthropogenic activities related to the construction project. In this sense, Table 6 presents a comparative estimate of the embodied CO_2_ for the concretes evaluated in this work. These estimations were obtained when adding the results obtained for each material by multiplying the mass with which this material participates in the mixture proportions by its corresponding embodied CO_2_. The results indicate that the substitutions of cement, silica fume, limestone powder, and limestone sand with vitrified clay led to a significant reduction of 170.32 kg CO_2_/m^3^ for the embodied CO_2_, representing a reduction of 27.2% for the green concrete without fibers and 20.2% for green concrete with fibers.

## 4. Conclusions

Based on the results obtained in this work, the following conclusions can be drawn.

The results of this work evidence the technical feasibility of a specific vitrified clay to be used in the production of ultra-high-performance concrete (UHPC), incorporating it as a substitute for cement (30%), silica fume (100%), and limestone sand-powders (30%), without detracting from the fresh stage properties, the compressive strength, the static modulus of elasticity, the flexural strength, and the flexural post-cracking residual strengths, and leading to significant reductions in the estimations of the embodied CO_2_.

Although the results and uncertainty indicators reported in this work were obtained for experimental laboratory tests, these constitute an exhortation for the industrial sector in charge of the precast of concrete elements to adopt it in its processes, since it is perceived as the sector that could benefit the most, being a sector in which rigorous procedures are commonly implemented for quality assurance, through which it would be expected that the variations in the results in relation to those obtained in this work would be minimal or negligible.

## Figures and Tables

**Table 1:** Properties of the particulate materials used to produce the UHPCs.

Materials	SG * g/cm^3^	WA %	SC %
CPO	3.11	---	---
SF	2.23	---	---
VC (D_50_= 8.4 μm)	2.61	0.0	---
VC (D_50_= 15.2 μm)	2.56	0.0	---
VC (D_50_= 40.7 μm)	2.48	1.8	---
VC (D_50_= 950 μm)	2.39	1.8	---
LS (D_50_= 950 μm)	2.70	1.5	---
LP (D_50_= 46.3 μm)	2.70	2.1	---
Superplasticizer	1.10	---	50
Defoamer	0.96	---	---
Steel Micro-fiber	7.90	---	---

* Dry basis. SG = specific gravity, WA = water absorption, SC = solids content.

**Table 2:** Particle size distribution of particulate materials.

Limestones and Vitrified Clay with D_50_ of								
PSD	CPO	SF	LS 950 μm	LP 46.3 μm	VC 8.4 μm	VC 15.2 μm	VC 40.7 μm	VC 950.0 μm
D_10_ (μm)	2.2	2.0	100.0	2.0	1.0	1.9	4.2	100.0
D_50_ (μm)	15.2	12.0	950.0	40.7	8.4	15.2	40.7	950.0
D_90_ (μm)	45.0	100.0	2400.0	100.0	33.8	141.2	112.0	2400.0

**Table 3:** Mixture proportions in kg/m^3^ for the UHPCs evaluated in this work, with the limestone or vitrified clay fractions in dry condition.

Materials	UHPC-C		UHPC-G	
	W/o SFR	With SFR	W/o SFR	With SFR
	kg/m^3^	kg/m^3^	kg/m^3^	kg/m^3^
Water	196.0	192.2	196.0	193.7
Superplasticizer	7.9	7.7	7.9	7.8
Air-Occluding	1.2	1.1	1.2	1.2
CPO	720.0	705.9	504.0	498.0
SF	80.0	78.4	0.0	0.0
VC (D_50_ = 8.4 μm)	0.0	0.0	80.0	79.1
VC (D_50_ = 15.2 μm)	0.0	0.0	216.0	213.4
VC (D_50_ = 40.7 μm)	0.0	0.0	126.2	124.7
VC (D_50_ = 950 μm)	0.0	0.0	234.3	231.6
LS (D_50_ = 950 μm)	868.8	851.8	608.2	600.9
LP (D_50_ = 46.3 μm)	467.9	458.7	327.5	323.6
Steel Micro-Fiber	0.0	154.9	0.0	156.1
Air content (%)	3.2	3.2	2.3	1.5

**Table 4:** Compressive strength development and standard deviation for a time window between 1 and 91 days.

Concrete	Compressive Strength (MPa) [Standard Deviation]						Slope (m, in MPa/Day)			
	1 d	3 d	7 d	28 d	56 d	91 d	1–7 d	7–28 d	7–91 d	28–91 d
UHPC-C	59 [0.6]	93 [1.1]	119 [0.7]	138 [8.0]	150 [5.9]	153 [1.4]	10.00	0.90	0.41	0.24
SFR-UHP-C	75 [6.6]	104 [1.3]	130 [0.6]	151 [0.1]	165 [2.3]	161 [2.1]	9.20	1.00	0.37	0.16
UHPC-G	45 [0.6]	78 [2.5]	98 [4.5]	128 [4.9]	153 [4.7]	162 [4.2]	8.80	1.40	0.76	0.54
SFR-UHPC-G	55 [0.1]	86 [2.5]	108 [1.3]	134 [4.2]	161 [1.3]	178 [1.1]	8.80	1.20	0.83	0.70

**Table 5:** Toughness indexes for several types of concretes [54,55,56].

Materials	Toughness Index (Arb. Units)			
	I_5_	I_10_	I_20_	I_r_
Plain Concrete	1	1	1	N.A.
Elastic-Plastic Material (E-P M)	5	10	20	N.A.
Fiber-Reinforced Concrete (FRC)	1–6	1–12	1–25	N.A.
UHPC	5.1–5.7	11.4–12.9	20.6–29.0	N.A.
SFR-UHPC-C	6.7	16.5	34.0	109.0
SFR-UHPC-G	6.2	14.9	34.2	110.8

N.A. No applicable.

**Table 6:** Estimation of embodied CO_2_ for the UHPCs evaluated in this work.

Items	Embodied CO_2_ [kgCO_2_/kg]	UHPC-C kgCO_2_/m^3^	SFR-UHPC-C kgCO_2_/m^3^	UHPC-G kgCO_2_/m^3^	SFR-UHPC-G kgCO_2_/m^3^	References
Superplasticizer	0.720	5.7	5.5	5.7	5.6	[58]
CPO	0.830	597.6	597.6	418.4	413.3	[59]
SF	0.000	0	0	---	---	[59]
VC (D_50_ = 8.4 μm)	0.040	---	---	3.2	3.16	[60]
VC (D_50_ = 15.2 μm)	0.040	---	---	8.6	8.5	[60]
VC (D_50_ = 40.7 μm)	0.020	---	---	2.5	2.5	[60]
VC (D_50_ = 950 μm)	0.003	---	---	0.7	0.7	[60]
LS (D_50_ = 950 μm)	0.002	1.7	1.7	1.2	1.2	[59]
LP (D_50_ = 46.3 μm)	0.017	8.0	7.8	5.6	5.5	[59]
Water	0.000	0.1	0.1	0.1	0.1	[59]
Air excluder	0.086	0.1	0.1	0.1	0.1	[59]
Steel fibers	1.490	0.0	230.8	0.0	232.6	[58]
Total (kg of CO_2_/m^3^)		613.1	843.6	446.1	673.3	

## Data Availability

The original contributions presented in the study are included in the article, further inquiries can be directed to the corresponding author.
